# Genome‐wide analysis of canonical Wnt target gene regulation in *Xenopus tropicalis* challenges β‐catenin paradigm

**DOI:** 10.1002/dvg.22991

**Published:** 2017-01-29

**Authors:** Yukio Nakamura, Stefan Hoppler

**Affiliations:** ^1^ Institute of Medical Sciences, Foresterhill Health Campus, University of AberdeenAberdeenAB25 2ZDUnited Kingdom

**Keywords:** Wnt signaling, β‐catenin, *Xenopus*, gastrula, ChIP‐seq, RNA‐seq

## Abstract

Wnt/β‐catenin signaling is an important cell‐to‐cell signaling mechanism that controls gene expression during embryonic development and is critically implicated in human diseases. Developmental, cellular, and transcriptional responses to Wnt signaling are remarkably context‐specific in different biological processes. While nuclear localization of β‐catenin is the key to activation of the Wnt/β‐catenin pathway and target gene expression, the molecular mechanisms of how the same Wnt/β‐catenin signaling pathway induces specific responses remain undetermined. Recent advances in high‐throughput sequencing technologies and the availability of genome information for *Xenopus tropicalis* have enabled us to uncover a genome‐wide view of Wnt/β‐catenin signaling in early vertebrate embryos, which challenges previous concepts about molecular mechanisms of Wnt target gene regulation. In this review, we summarize our experimental approaches, introduce the technologies we employed and focus on recent findings about Wnt target gene regulation from *Xenopus* research. We will also discuss potential functions of widespread β‐catenin binding in the genome that we discovered in this species.

## 1 Introduction

The Wnt/β‐catenin pathway is an important cell‐to‐cell signaling mechanism conserved among animals including humans (Loh, van Amerongen, & Nusse, 2016). Wnt/β‐catenin signaling function has been linked to a wide range of biological processes as diverse as embryonic axis formation and patterning, cell proliferation and differentiation, as well as tissue regeneration and cancer progression. Importantly, while Wnt/β‐catenin signaling acts through a single downstream nuclear effector called β‐catenin, the consequences of Wnt/β‐catenin signaling are remarkably context‐dependent, both in terms of tissue identity and stage of development. Studies on examples of individual genes regulated by Wnt/β‐catenin signaling had highlighted shared conserved molecular mechanisms of Wnt target gene regulation; however, a clear picture of how the same β‐catenin‐mediated Wnt signaling pathway can elicit such context‐specific responses has not yet been revealed.

*Xenopus* has served as a powerful model system to understand the fundamental molecular mechanisms of Wnt signaling that function universally in different tissues and animal systems. The unique experimental accessibility and size of the *Xenopus* embryo has also made it the model system of choice to apply recent advances in research technologies, such as high‐throughput sequencing in order to gain a genome‐wide view of target gene regulation by Wnt/β‐catenin signaling in early embryogenesis.

### 1.1 Nuclear β‐catenin is the hallmark of canonical Wnt signaling

**Figure 1:**
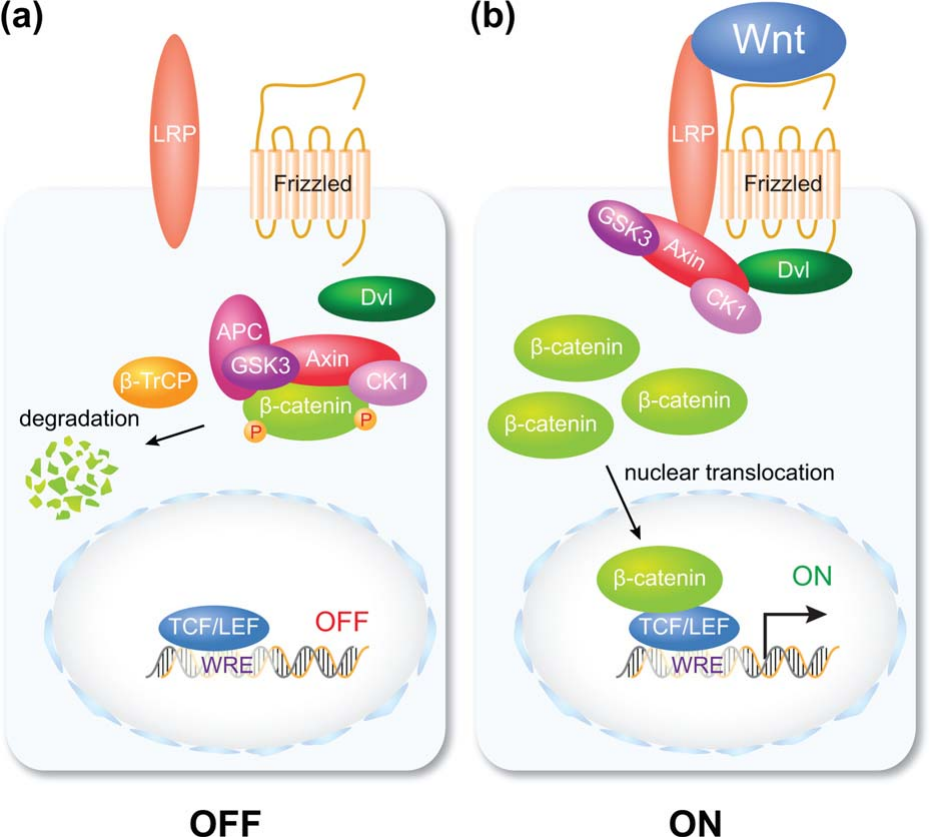
**Nuclear β‐catenin is the hallmark of canonical Wnt signaling.** Diagram depicting two cells and an outline of the canonical Wnt signal transduction pathway. (a) In a cell that is not responding to extracellular Wnt signaling any cytoplasmic β‐catenin protein is proteolytically degraded. (b) In response to extracellular Wnt signaling, any cytoplasmic β‐catenin protein is stabilized and enters the nucleus to regulate Wnt target gene expression in a complex with DNA‐binding transcription factors. For a more detailed recent review of Wnt signaling mechanisms, consult Hoppler and Nakamura (2014)

β‐catenin is central to the regulation of biological processes by so‐called canonical Wnt signaling. Without Wnt signaling activity β‐catenin is targeted for proteolytic degradation in the cytoplasm by a large multiprotein complex, termed the β‐catenin destruction complex (Figure 1a) (reviewed by Cadigan & Waterman, 2012; Hoppler and Nakamura, 2014). Once Wnt signaling is activated through interaction of Wnt ligands with Frizzled receptors and LRP co‐receptors at the cell surface, β‐catenin degradation is inhibited, which results in accumulation and translocation of β‐catenin to the nucleus (Figure 1b). Nuclear β‐catenin is consistently observed in a variety of experimental animal systems and cancer, where Wnt/β‐catenin signaling is active, and can thus be reliably used as the proxy (molecular readout) for active Wnt/β‐catenin signaling (reviewed in Hoppler & Moon, 2014; Nusse, He, van Amerongen, 2012).

**Figure 2:**
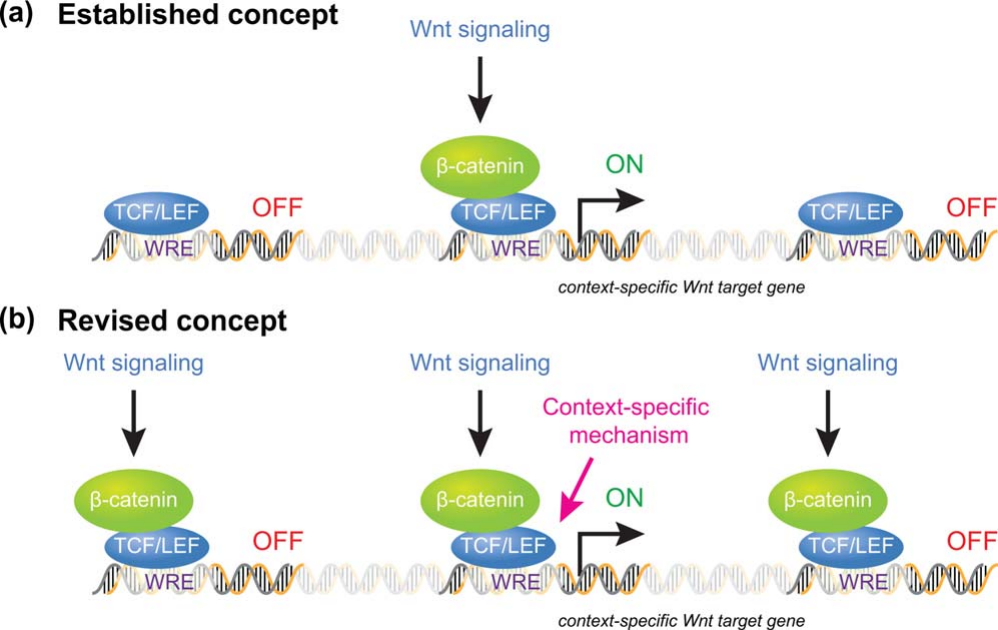
**Proposed mechanisms for regulating context‐specific Wnt target gene expression.** (a) The previously established paradigm asserted that transcription of context‐specific Wnt target genes is regulated by restricted access of nuclear β‐catenin protein to potential Wnt target gene sequences, with β‐catenin association to Wnt target gene sequences therefore considered both required and sufficient for context‐specific Wnt target gene regulation. (b) Genome‐wide analysis of context‐specific Wnt target gene regulation reveals more wide‐spread genome association of nuclear β‐catenin; including to Wnt target gene sequences that are not transcriptionally regulated in the particular cellular context studied. While β‐catenin association to Wnt target gene sequences is required, context‐specific mechanisms are additionally required for Wnt target gene transcription

In the nucleus, β‐catenin associates with particularly the TCF/LEF family of DNA‐binding transcription factors (reviewed by Hoppler & Waterman, 2014). The association of β‐catenin converts TCF/LEF proteins from a transcriptional repressor to a transcriptional activator, and the resulting β‐catenin/TCF complex activates Wnt target gene transcription (reviewed by Hoppler and Nakamura, 2014). The existing paradigm of Wnt/β‐catenin signaling therefore asserts that nuclear β‐catenin recruitment to target gene loci activates target gene expression (Figure 2a).

### 1.2 Maternal‐to‐zygotic transition in Wnt signaling in *Xenopus* embryos is ideal for studying context‐specific Wnt signaling mechanisms

**Figure 3:**
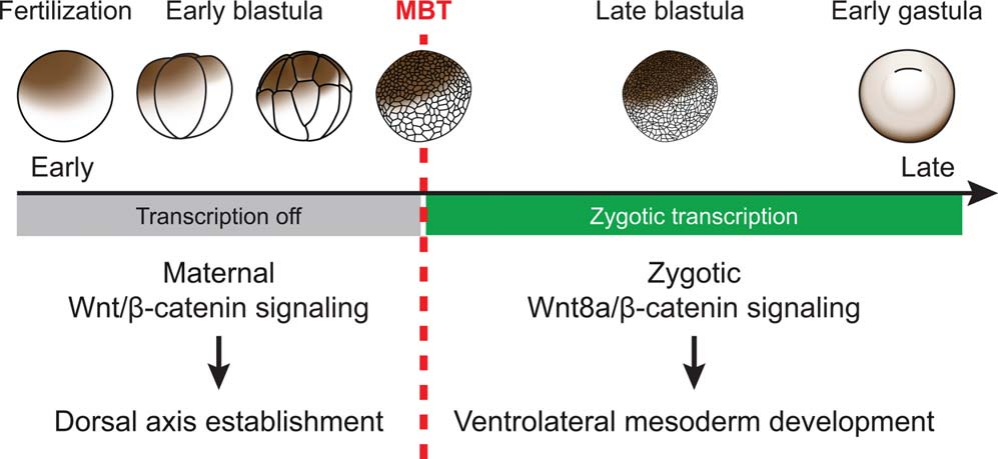
**Dramatic shift in response to Wnt signaling in early *Xenopus* embryogenesis.** Early *Xenopus* development is regulated by maternal gene products. Maternal Wnt signaling regulates dorsal axis establishment. After the MBT and the onset of zygotic transcription, Wnt signaling regulates essentially the opposite, ventral development or more precisely ventrolateral mesoderm development. Responses to maternal and to early zygotic Wnt signaling are mediated by essentially the same β‐catenin‐dependent signal transduction mechanism depicted in Figure 1

*Xenopus* embryos undergo a dramatic shift in the response to Wnt signaling early in development. This shift coincides with the onset of zygotic transcription at the mid‐blastula transition (MBT, Nieuwkoop‐Faber stage 8.5; Newport & Kirschner, 1982a, 1982b). Before the MBT, maternal Wnt signaling stabilizes maternally inherited β‐catenin in future dorsal cells (Larabell, Torres, Rowning, Yost, Miller, Wu, … Moon, 1997; Schneider, Steinbeisser, Warga, & Hausen, 1996; Schohl & Fagotto, 2002). This maternal β‐catenin sets up poised transcription of direct dorsal‐specific maternal Wnt target genes (Brannon, Gomperts, Sumoy, Moon, & Kimelman, 1997; Laurent, Blitz, Hashimoto, Rothbacher, & Cho, 1997; McKendry, Hsu, Harland, & Grosschedl, 1997), which then proceed with full transcription after the MBT (Blythe, Cha, Tadjuidje, Heasman, & Klein, 2010). The expression of these maternal Wnt target genes in dorsal blastomeres is responsible for establishing subsequent dorsal development (Figure 3).

In contrast, shortly after the MBT, the earliest zygotic Wnt signal (Wnt8a) promotes essentially the opposite developmental process, namely ventral development (Figure 3; Christian & Moon, 1993). Strikingly, both maternal and zygotic Wnt signaling use essentially the same β‐catenin‐dependent Wnt pathway (see Figure 1; Hamilton, Wheeler, & Hoppler, 2001). Therefore, the same β‐catenin‐mediated regulatory mechanism induces entirely different cellular responses within a short period of time early in *Xenopus* embryogenesis. This obvious contrast makes *Xenopus* embryos a unique model for dissecting the molecular mechanisms that determine context‐specific responses to Wnt signaling.

## 2 New Findings

### 2.1 Identification of direct zygotic Wnt target genes using high‐throughput sequencing in *Xenopus tropicalis*

Candidate gene approaches had previously led to the discovery of maternal Wnt‐regulated genes and of the molecular mechanisms controlling their expression (e.g. Blythe *et al*., 2010; Brannon *et al*., 1997; Nishita, Hashimoto, Ogata, Laurent, Ueno, Shibuya, & Cho, 2000). However, the genes that are regulated by zygotic Wnt8a signaling had been poorly understood. We therefore anticipated that identifying direct zygotic Wnt target genes and a comparison with maternal Wnt target gene regulation would be informative concerning the fundamental molecular mechanisms regulating context‐specific Wnt target gene expression.

The ideal features of *Xenopus tropicalis* for studying gene regulation and gene function compared to other vertebrate model systems include the ease of obtaining a large quantity of developmentally coordinated, relatively large sized embryos, and the rapid embryogenesis that facilitates analysis of different stages of development. Of more importance is recent progress in the availability of the genome sequence (Hellsten, Harland, Gilchrist, Hendrix, Jurka, Kapitonov, … Rokhsar, 2010) and the high‐throughput sequencing technologies coupled with the well‐established Chromatin Immuno‐Precipitation (ChIP) technique in this species (Akkers, Jacobi, & Veenstra, 2012). Together the *Xenopus tropicalis* model system has enabled us to embark on a genome‐wide analysis of zygotic Wnt target gene identification (Nakamura, de Paiva Alves, Veenstra, & Hoppler, 2016).

For the purpose of discovering direct zygotic Wnt8a/β‐catenin target genes, we logically defined two criteria for the genes that we wanted to identify: first, that the transcript levels of the genes must be altered (increased or decreased) when the Wnt8a signaling activity is experimentally changed; and second that there must be β‐catenin recruitment to genomic sequences nearby. To this end, we decided to gain a genome‐wide perspective by combining RNA‐seq transcriptome analysis using *wnt8a*‐depleted embryos and ChIP‐seq analysis of chromatin‐associated β‐catenin at early gastrula (post‐MBT).

### 2.2 RNA‐seq analysis identifies genes regulated by zygotic Wnt8a signaling

**Figure 4:**
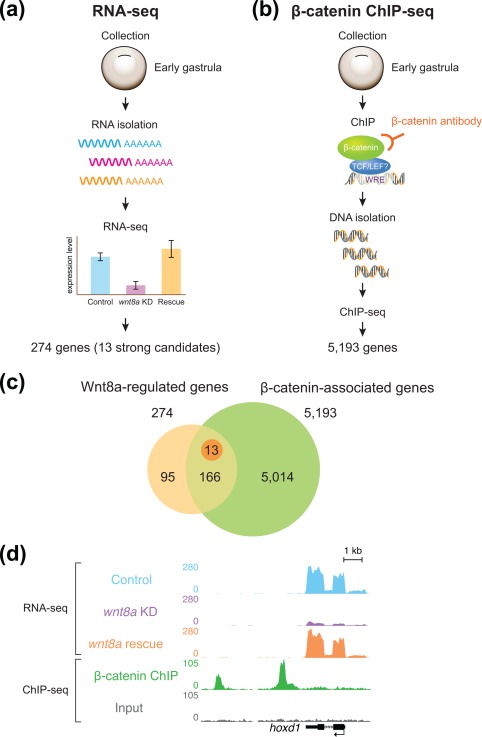
**Identification of direct context‐specific Wnt/β‐catenin target genes in a genome‐wide approach.** (a) Transcriptome analysis (with RNA‐seq) of early gastrula‐stage embryos. Comparing the transcriptome of control embryos with embryos with *wnt8a* knockdown and those with experimentally reinstated Wnt8a identifies genes regulated by Wnt8a signaling in post‐MBT embryos. (b) ChIP‐seq analysis with a β‐catenin‐specific antibody identifies DNA‐sequences associated with β‐catenin and nearby potentially regulated genes in early gastrula‐stage embryos. (c) Venn diagram comparing Wnt8a‐regulated genes (in beige) from the RNA‐seq analysis (see panel A) with the β‐catenin associated genes (in green) from the ChIP‐seq analysis (see panel B) with the overlap or intersection identifying direct Wnt8a target genes. Note that there are many more identified β‐catenin associated genes than identified Wnt8a signaling‐regulated genes in early gastrula embryos. (d) Example of a direct Wnt8a‐target gene (*hoxd1*) in genome view, with from top to bottom, transcripts from control embryos, transcripts from the *wnt8a* knock down (note reduced expression), transcripts from embryos with experimentally reinstated Wnt8a expression, β‐catenin‐associated DNA sequences (“β‐catenin” peaks), sequences of the control sample of the ChIP‐seq experiment (Input control) and the *hoxd1* gene model. Note β‐catenin‐associated DNA sequences downstream (left) of the rest of the *hoxd1* gene and transcript sequences of particularly the exon sequences in the control and *wnt8a* rescue samples

For our RNA‐seq study, we developed an experimental design to reliably identify genes regulated by Wnt8a signaling. Not only did we compare the transcriptomes between control embryos and *wnt8a*‐depleted embryos (by *wnt8a* morpholino (MO) knock down), we also studied gene expression profiles of the embryos, in which the *wnt8a* knock down was experimentally rescued with injection of a MO‐insensitive *wnt8a*‐expressing DNA construct (Figure 4a, see also Christian & Moon, 1993). This approach successfully ruled out possible false‐positives resulting from the unexpected effect of MO injection and allowed the reliable selection of genes whose expression is regulated by zygotic Wnt8a signaling.

Our bioinformatics analysis of the resulting RNA‐seq data led to the identification of about 13 clear Wnt8a‐regulated genes (Figure 4a) and a further 274 statistically more loosely supported potential Wnt8a‐regulated genes (for a detailed list see Nakamura *et al*., 2016). The list of these genes included, as expected, previously identified Wnt target genes [*sp5* (Weidinger, Thorpe, Wuennenberg‐Stapleton, Ngai, & Moon, 2005), *hoxd1* (Janssens, Denayer, Deroo, Van Roy, & Vleminckx, 2010), and *msgn1* (Wang, Li, Chen, & Ding, 2007)], a number of feed‐back Wnt signaling component genes (*fzd10* and *xarp/axin2*); but importantly, none of the known maternal Wnt target genes, highlighting robust mechanisms that exclude them in order to ensure correct context‐specific responses to Wnt signaling.

### 2.3 ChIP‐seq analysis identifies genomic loci directly targeted by β‐catenin in the post‐MBT embryo

ChIP‐seq uses the Chromatin Immuno‐Precipitation (ChIP) technique combined with high‐throughput sequencing that enables us to obtain a genome‐wide snapshot of protein–DNA interactions in a particular context (e.g., cells, stages, etc). ChIP‐seq analysis turned out to be more challenging than RNA‐seq approaches, as its success highly depends on the availability of a high quality antibody targeting the protein of interest, and on the quantity of chromatin‐bound proteins. In particular, because *Xenopus* embryos are yolk‐rich, longer fixation times were required and thus additional optimization was necessary for determining appropriate chromatin crosslinking time depending on the stage of development.

β‐catenin ChIP‐seq (Figure 4b) is a powerful approach previously used for identifying direct Wnt/β‐catenin targets in defined specific contexts, such as embryonic stem cells and cancer tissue (Bottomly, Kyler, McWeeney, & Yochum, 2010; Park, Ma, O'brien, Chung, Guo, Cheng, … McMahon, 2012; Schuijers, Mokry, Hatzis, Cuppen, & Clevers, 2014; Watanabe, Biesinger, Salmans, Roberts, Arthur, Cleary, … Dai, 2014). After extensive optimization and validation, our β‐catenin ChIP‐seq revealed over 10,000 β‐catenin‐associated sequences along the *Xenopus tropicalis* genome, which were bioinformatically linked to approximately 5,000 nearby genes (representing approx. 25% of all predicted *Xenopus tropicalis* genes). A high degree of correlation between those many β‐catenin‐associated sequences with genomic locations previously identified as regulatory sequences (e.g. promoters and enhancers) confirmed this β‐catenin chromatin‐association to be due to specific binding.

### 2.4 β‐catenin recruitment is not the hallmark of Wnt‐mediated transcriptional regulation

Our combined analysis revealed that the number of β‐catenin‐associated gene loci is considerably larger than that of Wnt‐regulated genes in post‐MBT embryos (Figure 4c). Those Wnt8a‐regulated genes that we tested were overwhelmingly found to be correlated with nearby β‐catenin‐associated sequences (e.g., *hoxd1*, Figure 4d); and β‐catenin‐association to these sequences was as expected regulated by Wnt8a signaling. Thus while Wnt signaling‐regulated β‐catenin association proved to be required for transcriptional regulation of direct target genes; the results of our analysis implied that β‐catenin association with genomic sequences may not be sufficient for transcriptional regulation. The observed widespread recruitment of β‐catenin along the genome is surprising, because nuclear β‐catenin binding to chromatin was believed to be the established hallmark for transcriptional activation of nearby genes by Wnt signaling (see Figures 1 and 2a).

Our conclusions further became firm after subsequent analysis comparing β‐catenin recruitment to context‐specific genes for the different contexts before and after the MBT. We experimentally increased or decreased the Wnt8a signaling activity in post‐MBT embryos and studied transcription levels of the “wrong” genes, i.e., of maternal Wnt‐regulated genes that were uncovered to have β‐catenin recruitment by our β‐catenin ChIP‐seq analysis in post‐MBT embryos. In these post‐MBT experimental conditions, as expected, the transcription of these maternal Wnt target genes remained unaffected. However, the β‐catenin binding level was significantly increased by the enhanced Wnt8a activity, demonstrating that Wnt‐regulated β‐catenin recruitment does not necessarily lead to transcriptional activation of associated genes. This finding further prompted us to test whether maternal β‐catenin also has any potential to control direct zygotic Wnt8a target genes before the MBT. Interestingly, we observed that maternal β‐catenin was precociously associated with zygotic Wnt8a target gene loci in pre‐MBT embryos; that this β‐catenin association was regulated by maternal Wnt signaling even though as expected there was no transcriptional regulation of these zygotic Wnt8a target genes. Taken together, these findings clearly show that Wnt‐regulated β‐catenin recruitment is not sufficient for transcriptional activation in the “wrong” developmental context (see Figure 2b).

### 2.5 Context‐specific regulation of zygotic Wnt8a target genes

What is so special about the transcriptionally regulated Wnt8a/β‐catenin target genes? Since direct maternal Wnt/β‐catenin target genes are regulated by combinatorial Wnt and Activin/Smad2 signaling (Crease, Dyson, & Gurdon, 1998; Nishita *et al*., 2000), we hypothesized that an analogous shared tissue‐specific molecular regulatory mechanism would control Wnt8a target gene regulation.

We however uncovered that rather than sharing a single ventral mesoderm‐specific mechanism, Wnt8a target genes turned out to have more gene‐specific regulatory mechanisms. For some of the Wnt8a target genes, BMP signaling is indispensable, and for some others FGF signaling is essential. Analysis of expression domains showed that BMP‐ or FGF‐dependent Wnt8a target genes are expressed in different tissues of early gastrulae. Overexpression and inhibition experiments demonstrated that these signaling pathways provide spatially different, partially overlapping contexts for Wn8a target genes to respond to Wnt8a/β‐catenin signaling.

Although the BMP or FGF signaling pathways are required for Wnt8a target gene expression, neither BMP nor FGF signaling controls β‐catenin activation or accessibility to chromatin. The context‐specific Wnt target gene regulation by these pathways occurs separately from β‐catenin recruitment (see Figure 2b).

The detailed molecular mechanisms of this context‐specific Wnt target gene regulation remain unclear. So far, a number of β‐catenin‐interacting proteins have been reported (Mosimann, Hausmann, & Basler, 2009; Stadeli, Hoffmans, & Basler, 2006). These proteins are recruited to regulatory DNA sequences by β‐catenin and are involved in transcriptional regulation of Wnt/β‐catenin target genes. BMP and FGF signaling may regulate the recruitment of these proteins to β‐catenin in early *Xenopus* development. Alternatively, these co‐regulating BMP and FGF pathways may more independently influence transcriptional activation of Wnt/β‐catenin target genes by promoting the removal of any negative regulators or the addition of positive regulators to separate cis‐regulatory sequences of the same gene.

## 3 Discussion

### 3.1 Biological function of widespread β‐catenin recruitment to the genome

As noted above, our β‐catenin ChIP‐seq has revealed β‐catenin recruitment to a large number of genomic regions, which vastly exceed the number of Wnt8a‐regulated genes in this context. Genome‐wide binding outside of direct transcriptional regulation has been observed for other transcription factors and in different systems (MacQuarrie, Fong, Morse, & Tapscott, 2011). As ChIP‐seq is becoming used more generally in various systems, such extensive binding is becoming emphasized and it becomes important to understand any possible biological significance. In analogy to DNA‐binding transcription factors, some β‐catenin association to the chromatin might be non‐functional (cf. Li, MacArthur, Bourgon, Nix, Pollard, Iyer, … Biggin, 2008), or it could be relevant for several previously proposed functions (cf. MacQuarrie *et al*., 2011):

- The first proposal involves binding of a transcription factor through specific DNA motifs but this is not immediately associated with direct transcription at nearby sites. Only at later stages or in other tissues does this binding lead to expression of those genes when it is integrated with binding by additional transcription factors. Interestingly, our β‐catenin‐binding sites included many genes activated at later stages by Wnt signaling [e.g., *neurod1* (Kuwabara, Hsieh, Muotri, Yeo, Warashina, Lie, … Gage, 2009) and 70% of β‐catenin‐bound genes of a colorectal cancer cell line (Watanabe *et al*., 2014)], suggesting that β‐catenin binding in early gastrulae could be a precocious event that can only act cooperatively with subsequent cooperative regulation at later stages.
- Another proposal involves a buffering system through binding to many relatively accessible sites in the chromatin (cf. Lin and Riggs, 1975). In this mechanism, binding that is not related to transcription is employed to limit the availability of unbound transcription factors, thereby fine‐tuning direct specific gene expression. As demonstrated, Wnt signaling activation leads to high levels of nuclear β‐catenin. Considering this fact, extensive β‐catenin recruitment might be attributed to part of this buffering system to prevent inadvertent strong transcriptional activation.
- A further proposal involves chromatin reorganization. Pioneer transcription factors can also bind to otherwise inaccessible sites of closed chromatin (Iwafuchi‐Doi & Zaret, 2016). Binding of these pioneer factors induces recruitment of chromatin remodelers or modifiers, which in turn results in genome‐wide or more regional histone modifications to make chromatin more accessible to other transcription factors (Cao, Yao, Sarkar, Lawrence, Sanchez, Parker, … Tapscott, 2010; Knoepfler, Zhang, Cheng, Gafken, McMahon, & Eisenman, 2006). Indeed, β‐catenin and TCF7L2 recruitment to chromatin was reported to induce histone acetylation in a widespread region by recruiting CBP, a histone acetyltransferase; yet this recruitment of β‐catenin is independent of transcription (Parker, Ni, Chang, Li, & Cadigan, 2008). Interestingly, our identified β‐catenin peaks show a strong correlation with both histone acetylation and p300 occupancy, a paralog of CBP. Thus, our discovered β‐catenin recruitment at a large number of sites could be involved in such chromatin reorganization, rather than causing direct transcription.

Genome‐wide β‐catenin recruitment could be involved in some of these proposed molecular mechanisms. However, it is noted that β‐catenin is not a DNA‐binding transcription factor. Differences in the contribution of β‐catenin binding could change depending on the DNA‐binding transcription factors that β‐catenin interacts with. Generally, TCF/LEF is the main partner of β‐catenin for recruitment to chromatin (reviewed by Hoppler & Waterman, 2014). Other studies also suggested the potential interaction of β‐catenin with other DNA‐binding transcription factors (reviewed by Cadigan and Waterman, 2012), which is consistent with our findings (for more detail consult Nakamura *et al*., 2016). To clarify and define what processes β‐catenin recruitment is involved in, it will be necessary to identify what factors induce β‐catenin recruitment. Further ChIP‐seq analysis of those transcription factors together with β‐catenin in samples where those proteins are knocked down or removed will be required, and *Xenopus* will again be ideal for such studies.

## 4 Conclusions and perspective

Conventional research on Wnt signaling has logically focused on identifying mechanisms that are universally used in different contexts. However, our understanding is still limited regarding why certain Wnt target genes remain transcriptionally refractory to Wnt signaling activation in one context but can be activated by Wnt signaling in another context.

Nuclear β‐catenin recruitment has been considered an instant link to transcriptional activation of Wnt target genes. However, the finding of widespread β‐catenin recruitment in the *Xenopus* genome has challenged this concept and brought about a new paradigm for a Wnt‐regulated transcriptional mechanism. This new paradigm is not only relevant to embryogenesis but also to regeneration and cancer, as well as other Wnt‐related diseases, where a change in cellular contexts may induce expression of genes with precocious β‐catenin binding. The observed widespread β‐catenin binding may also contribute to yet undetermined functions, such as chromatin reorganization and signaling buffering mechanisms. Further investigation of context‐specific Wnt target gene regulation and the role of widespread β‐catenin recruitment in the genome will further benefit from the use of new technologies, such as genome editing, combined with now well‐established high‐throughput sequencing approaches in the experimentally accessible *Xenopus* model system.
